# CD133 Expression in Glioblastoma Multiforme: A Literature Review

**DOI:** 10.7759/cureus.3439

**Published:** 2018-10-11

**Authors:** Syed Ijlal Ahmed, Gohar Javed, Altaf Ali Laghari, Syeda Beenish Bareeqa, Saba Farrukh, Shajeeah Zahid, Syeda Sana Samar, Kashif Aziz

**Affiliations:** 1 Neurosurgery, Liaquat National Hospital and Medical College, Karachi, PAK; 2 Neurosurgery, The Aga Khan University, Karachi, PAK; 3 Oncology, Jinnah Medical and Dental College, Karachi, PAK; 4 Internal Medicine, Liaquat National Hospital and Medical College, Karachi, PAK; 5 Internal Medicine, Jinnah Sindh Medical University, Karachi, PAK; 6 Internal Medicine, Icahn School of Medicine at Mount Sinai/Queens Hospital Center, New York, USA

**Keywords:** glioblastoma multiforme (gbm), cd133, brain tumor

## Abstract

Glioblastoma, also known as glioblastoma multiforme (GBM), is the most common primary brain tumor. Extensive research has been carried out to discover the factors associated with the course and progression of GBM. CD133 is a glycoprotein antigen found in normal and malignant tissues. CD133 has been recognized as a marker for the growth of cancer cells. The association between this tissue marker and GBM is being investigated. The aim of this review was to evaluate the role of CD133 as a tumor marker for the prognosis of GBM.

## Introduction and background

Malignant gliomas are the most frequent and lethal tumors originating in the central nervous system (CNS). The most biologically aggressive subtype is glioblastoma multiforme (GBM) [World Health Organization (WHO) grade IV astrocytoma], a tumor associated with a dismal prognosis [1].

The Central Brain Tumor Registry of the United States (CBTRUS) statistical report on the primary brain and CNS tumors diagnosed in the US in 2006-2010 stated that the age-related occurrence of GBM was 3.19/100,000 population. Data also showed that the incidence of GBM was the highest among the brain and CNS tumors that are considered lethal and malignant [2].

The median age for people diagnosed with GBM is 64 years and they are the most susceptible [3]. GBMs in children are infrequent and account for very few cases. Between the age range of one to 19 years, only three percent of such brain- and CNS-related tumors have been reported. As previously stated, there is a higher probability of occurrence of these tumors with increasing age. The maximum incidence is observed at age 75 to 84 years but then reduces in the following years. Since there has been an increase in average life expectancy, a rise in the number of such tumors is inevitable [4].

Stem cell biology has helped us understand many aspects of tumorigenesis and in particular, the complexity of cancers. The discussion on cancers would not be replete without mentioning the stem cells [5-7]. Research has shown that stem cells may play a central role in the malignancies.

Stems cells are progenitor cells and have self-renewal ability. This property, if left extensive and uninhibited, can lead to the proliferation of such cells in the form of malignancies. They also have the ability to divide into multiple lineages [8-9]. This again indicates their role in a number of malignancies.

A small populace of cancer stem cells (CSC) has been discovered, which may prove to be associated with GBM. These CSCs express an antigen called CD133, also known as prominin-1. This antigen is expressed as a cell membrane glycoprotein in humans. It is encoded by the PROM1 gene, and evidence suggests that it can be used as a biomarker for some tumors [10].

Although CD133 has not been acknowledged globally as a marker for CSCs, transcriptomic profiling of certain cells based on the CD133 protein have helped develop better insights into tumorigenesis. CD133 is not only a biomarker for segregation and characterization of stem cells but may also have a role in cell growth, proliferation and pathophysiology of the growing tumors [11].

This topic can be extended to GBM as well. The expression of CD133 on such tumors and its impact on the outcome can be studied.

## Review

A classical example of the in-depth examination and understanding of the CSCs in solid tumors is GBM. Evidence suggests that GBM can result from various cancer stem cells. This was evaluated by analyzing the gene expression profiles of 17 GBM CSC lines. Using a 24-gene signature, the GBMs were categorized into two sub-groups. The first sub-group was type I CSC lines that displayed “pro-neural” signature genes and resembled the fetal neural stem cells. Type II CSC lines displayed “mesenchymal” transcriptional profiles that resembled the adult neural stem cells. Type I CSCs were CD133 positive and showed adherent growth and therefore formed neurospheres. However, type II CSC lines were CD133 negative and did not show adherent growth and therefore were less likely to form neurospheres [12].

Another study analyzed data in three cultured cell lines taken from the GBM patients regarding the percentage of cells that were CD133 positive and negative. On observation, some traits were distinctly linked to those tissues that expressed CD133. A distinctive cancerous property to inhibit programmed cell death was exhibited by CD133-positive cells. Their inhibition of apoptosis was supported by the activation and increase in the levels of mRNA [13]. CD133-positive cells were resistant to chemotherapeutic agents such as temozolomide, carboplatin, paclitaxel (taxol) and etoposide. Contrarily, this property was absent in the CD133-negative cells. On pursuing this study, it was further noted that CD90 and CD44 are the markers associated with neural precursors whose average mRNA levels increased exponentially in the CD133-positive cells as opposed to the CD133-negative cells [10].

Other studies analyzed the CD133-positive cells in GBM. A study used highly purified GBM CD133+/- cells to cross-examine profiles that were derived from tumor samples with significant mass. CD133-positive cells showed evidence of hypermutated and highly malignant sub-types of GBM [14].

In addition, different studies and investigations on other tumors have shown that CD133 closely parallels with the size of the tumors, a worse prognosis and an increase in the lymph node metastasis [13,15]. However, exposing CD133 to AC133 [16], which is an antibody to CD133, causes the inhibition of cancerous growth, metastasis and spheroid-forming capacity, thus inhibiting the oncogenic potential. It decreases the expression of CD133, thereby causing regression of the tumor [17-18].

A notable method to establish the expression of CD133 in GBM by way of its suppression. Clinical trials carried out in lab rats showed that CD133 suppression resulted in a significant increase in the number of mice that survived the tumor and eventually showed its absence [19-21]. It was also observed that the cells that suppressed CD133 using a shRNA/hairpin vector (is an artificial RNA molecule with a tight hairpin turn that can be used to silence target gene expression via RNA interference) formed fewer colonies and displayed lack of self-renewal [18,22]. The neurospheres formed by the silenced cells were also smaller and showed reduced spheroid-forming capacity. However, no difference was seen or observed between the silenced and non-silenced cells in terms of the percentage of cells that carried out apoptosis [20,23].

Conclusively, there was a marked reduction in the self-proliferation and growth of cells with the reduction of CD133 expression [24-25].

A meta-analysis tackled the debate of CD133 being a significant prognostic factor in GBM. The prognostic role of CD133 in GBM patients was evaluated. Publications assessing the prognostic significance of CD133 expression in GBM patients were identified in PubMed, Embase and Web of Science up to November 2014 [26]. Ten studies with a total of 715 GBM patients were included in the meta-analysis. Overall, high CD133 expression was associated with poor overall survival in patients with GBM.

Another observation that should also be brought to light is that several studies have shown that some tissues isolated from the CD133-negative GBM patients were capable of converting themselves into CD133-positive cells and therefore, exhibiting high malignancy and rapid tumor progression. 

Several studies demonstrate the importance of CD133 expression on the plasma membrane as compared to the cytoplasm. As stated previously, CD133 is expressed as a cell membrane protein. However, there is also evidence of an intracellular collection of CD133 [27-28]. Hence, the isolated tissues expressing a high amount of membrane-bound CD133 showed greater tumorigenic potential and the ability to form clones as compared to the cells with the presence of cytoplasmic CD133. Conclusively, cells that exhibit CD133 on the cell surface substantially increase the self-renewal rate and act as potent tumor initiators [22]. However, some studies suggested that the CD133-negative cells that are derived from the neurospheres of GBM patients have a self-renewal ability that increases the malignant potential [29].

## Conclusions

Following extensive procedures, using both quantitative and qualitative methods of research, it can be deciphered that the greater the expression of CD133 in GBM patients, the more the malignancy potential of the cells. In light of literature review, it is evident that CD133 is important for the malignant oncogenic potential of GBM stem cells as its silencing hinders both self-renewal and tumorigenic capacities of the GBM stem cells. However, a slightly contradictory evidence shows that some CD133-negative cells can also develop aggressive malignancies.
